# Acute myeloid leukemia with inv(16)(p13.1q22) and deletion of the 5’MYH11/3’CBFB gene fusion: a report of two cases and literature review

**DOI:** 10.1186/s13039-020-0474-9

**Published:** 2020-01-30

**Authors:** Lili Lv, Jingwei Yu, Zhongxia Qi

**Affiliations:** 1 Department of Oncology and Hematology, The Second Hospital of Jilin University, Changchun, Jilin, China; 2 Department of Laboratory Medicine, University of California San Francisco, San Francisco, CA USA

**Keywords:** AML with inv(16), Deletion of 5’*MYH11/*3’*CBFB*, SNP microarray

## Abstract

**Background:**

Abnormalities of chromosome 16 are found in about 5–8% of acute myeloid leukemia (AML). The AML with inv(16)(p13.1q22) or t (16;16)(p13.1;q22) is associated with a high rate of complete remission (CR) and favorable overall survival (OS) when treated with high-dose Cytarabine. At the inversion breakpoints, deletion of 3’*CBFB* has been reported, but most of them were studied by chromosome and fluorescence in situ hybridization (FISH) analyses. The genomic characteristics of such deletions remain largely undefined, hindering further understanding of the clinical significance of the deletions.

**Case presentation:**

We report here two AML cases with inv(16) and deletion of the 5’*MYH11*/3’*CBFB* gene fusion, which were characterized by chromosome, FISH, and single nucleotide polymorphism (SNP) microarray analyses. Both cases have achieved CR for more than three years.

**Conclusions:**

Deletion of 3’*CBFB* in AML with inv(16) is also accompanied with deletion of 5’*MYH11* in all the cases studied by SNP microarray, suggesting that 3’*CBFB* and 5’*MYH11* were most likely deleted together as a fusion product of inv(16) instead of occurring separately. In concert with the findings of other published studies of similar patients, our study suggests that deletion of 5’*MYH11*/3’*CBFB* in AML with inv(16) may not have negative impact on the prognosis of the disease.

## Background

Abnormalities of chromosome 16 are found in about 5–8% of acute myeloid leukemia (AML) and are one of the three AML defining chromosomal aberrations regardless of blast percentage under the World Health Organization (WHO) classification [1]. The AML with inv(16)(p13.1q22) or t (16;16)(p13.1;q22) is associated with a high rate of complete remission (CR) and favorable overall survival (OS) when treated with high-dose Cytarabine [2, 3]. The inv(16) results in a leukemogenic *CBFB/MYH11* gene fusion [4, 5]. However, additional chromosome changes and/or gene mutations, such as + 22, + 8, deletion of 7q, and the *CBL*, *FLT3*, *KIT* gene mutations, are frequently found in AML with inv(16). These additional changes/mutations may influence the OS positively or negatively [6, 7]. For example, gain of an additional chromosome 22 in AML with inv(16) may predict an improved outcome [6, 8], whereas *KIT* mutations appear to have an increased risk of relapse and shorter survival in adult patients [6, 9]. Less commonly, deletions, especially deletion of 3’*CBFB,* may occur at the inversion breakpoints*.* Fourteen cases with inv(16) and deletion of 3’*CBFB* have been reported in literature [10–19]. However, only three of them were studied by microarray analysis, and only one was reported with unambiguous breakpoint coordinates [10]. The genomic characteristics of the vast majority of the reported *3’CBFB* deletions were undefined.

Here we reported two AML with inv(16) cases, both carried an additional deletion at one inversion breakpoint involving the fusion between 5’*MYH11* at 16p13.1 and 3’*CBFB* at 16q22. The genomic characteristics of both cases were characterized by chromosome, FISH and SNP microarray analyses. We also reviewed similar cases reported in literature to investigate possible clinical significance of the deletions at the inv(16) breakpoints.

## Case presentation

### Case 1

A 24-year-old male presented with intermittent fever and sore throat. A complete blood examination demonstrated a hemoglobin (Hb) count of 70 g/L, a white blood cell (WBC) count of 170 × 10^9^/L with 80% blasts and a platelet count of 25 × 10^9^/L. He had no hepatosplenomegaly. His bone marrow (BM) aspirate exhibited greater than 90% myelomonoblastic cells, with increased maturing eosinophils. Cytochemical staining was positive for peroxidase and esterase. Flow cytometry showed 76% positive CD34 and 5% positive CD64, but negative CD14, consistent with a diagnosis of AML. The patient started induction chemotherapy and achieved a complete hematological recovery on day 21. He then received autologous stem cell transplant (ASCT) after two cycles of consolidation chemotherapy and has remained in CR for three years.

### Case 2

A 47-year-old male presented with intermittent low-grade fever and progressive fatigue. A complete blood examination demonstrated a Hb count of 92 g/L, a WBC count of 3.5 × 10^9^/L and a platelet count of 43 × 10^9^/L. This patient had splenomegaly. His BM aspirate showed 50% myeloid blasts. Flow cytometry showed 50% blasts expressed CD34, CD117, CD13, CD33, HLA-DR, and myeloperoxidase (MPO), consistent with a diagnosis of AML. The patient started induction chemotherapy and achieved a complete hematological recovery. He then received two cycles of consolidation chemotherapy. The patient has been in CR for four years.

## Methods

### Chromosome and FISH analyses

Chromosome G-banding was performed following standard techniques on BM aspirate. FISH was carried out with the commercial *CBFB* dual-color break-apart probe kit (Abbott Laboratories, Lake Bluff, IL) following the manufacturer’s protocol. The results were analyzed using the Leica CytoVision system (Leica Biosystems, San Jose, CA).

### SNP microarray analysis

Genomic DNA was extracted from BM using QIAGEN EZ1 kit (Qiagen, Hilden, Germany). SNP microarray was set up using Illumina CytoSNP-850 K v1.1 BeadChip (Illumina, San Diego, CA) and analyzed using BlueFuse Multi (Illumina), based on human genome build GRCh37/hg19.

The nomenclature for the chromosome, FISH and array findings is based on the International System for Human Cytogenomic Nomenclature (ISCN) 2016 [20].

## Results

**Fig. 1:**
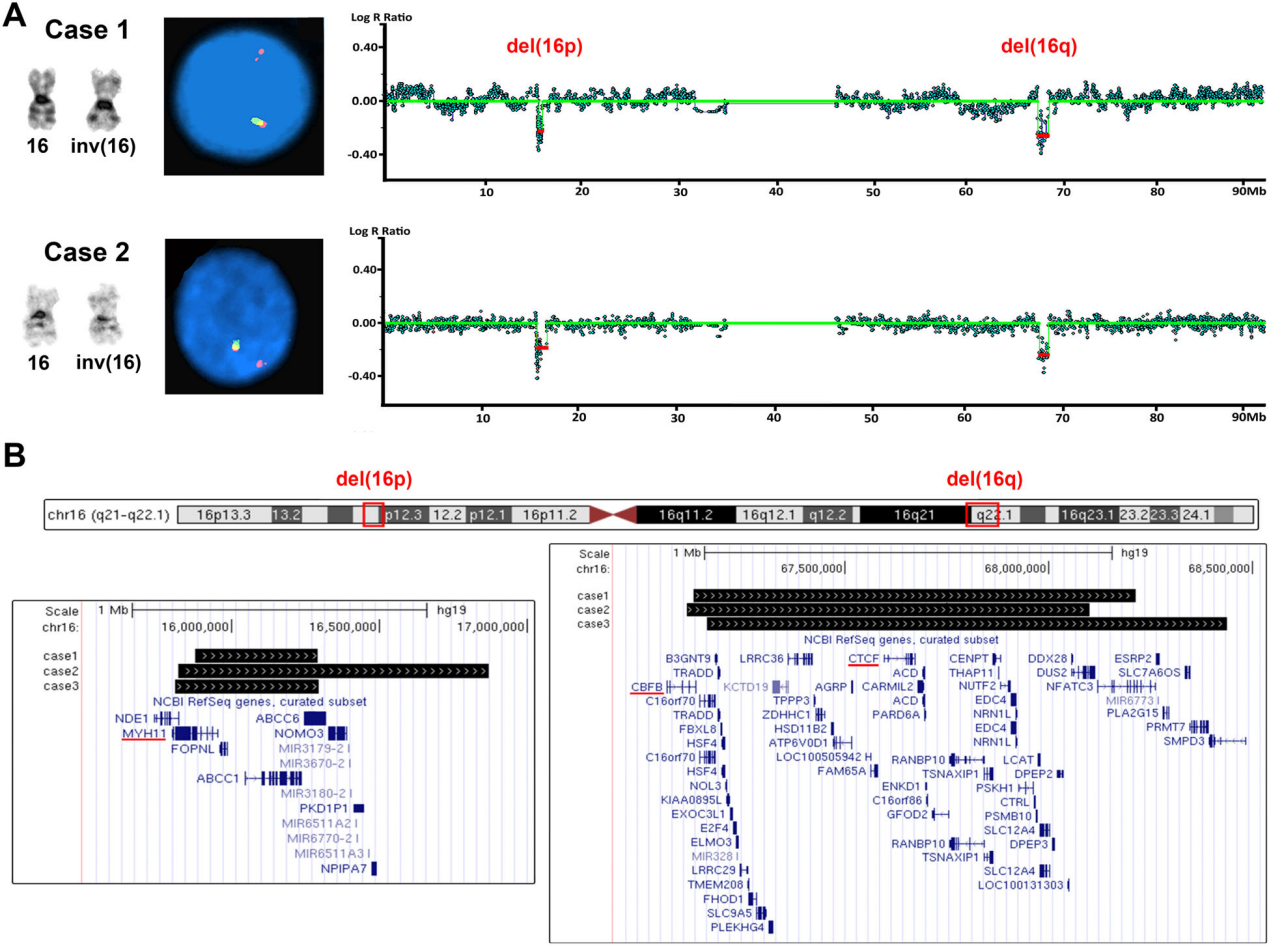
**a** Chromosome 16 with inv(16)(p13.1q22), 3’*CBFB* deletion detected by interphase FISH (5’*CBFB*, red; 3’*CBFB*, green), and two small deletions on chromosome 16 detected by SNP microarray. **b** Genes in the deleted regions (black bars), adopted from UCSC genome browser (https://www.genome.ucsc.edu). Case 3 was from Dawson et al. [10], and COSMIC cancer genes were underlined

In case 1, chromosome analysis detected an abnormal karyotype with a pericentric inversion of chromosome 16 in all 20 cells analyzed (Fig. 1a), consistent with the diagnosis of AML with inv(16)(p13.1q22); *CBFB*-*MYH11*. In addition, 4 of these cells showed an extra copy of chromosome 22, which is a common secondary change in this disease. FISH analysis confirmed the inversion but also detected a deletion of 3’*CBFB* in approximately 95.5% of the interphase cells examined (Fig. 1a). SNP microarray confirmed the gain of chromosome 22 and a 1.1 Mb deletion involving 3’*CBFB* on the long arm of chromosome 16 within 16q22.1, and further detected a 416 Kb deletion involving the 5*’MYH11* gene on the short arm of chromosome 16 within 16p13.11. The 16q22.1 deletion involved 53 known genes including 3’*CBFB*, and the 16p13.11 deletion involved 7 known genes including 5*’MYH11* (Fig. 1b). The nomenclature of the cytogenomic findings in case 1 can be described as: 46,XY,inv(16)(p13.1q22)[16]/47,idem,+ 22[4].nuc ish (5’CBFBx2,3’CBFB×1)(5’CBFB con 3’CBFB×1)[191/200].arr[GRCh37] 16p13.11(15875744_16291983)×1[0.9],16q22.1(67128019_68214140)×1[0.9],(22) ×3[0.8].

In case 2, a similar inversion of 16 with gain of additional chromosomes 9 and 22 was detected in all 20 metaphases analyzed by chromosome analysis (Fig. 1a). FISH revealed a similar abnormal signal pattern with a deletion of 3’*CBFB* in approximately 83.5% of the interphase cells examined (Fig. 1a). Similar to case 1, SNP microarray confirmed the chromosome and FISH findings and detected an additional deletion on the short arm of chromosome 16 (Fig. 1a). The 16p13.11p12.3 deletion is 1.1 Mb in size involving 14 known genes including 5’*MYH11*, and the 16q22.1 deletion is 986 kb in size involving 52 genes including 3’*CBFB* (Fig. 1b). The cytogenomic findings of case 2 can be described as: 48,XY,+ 9,inv(16)(p13.1q22),+ 22[20].nuc ish(5’CBFBx2,3’CBFB×1)(5’CBFB con 3’CBFB×1) [167/200].arr[GRCh37] (9)×3[0.7],16p13.11p12.3(15817490_16869754)×1[0.7],16q22.1(67113418_68099821)×1[0.7],(22)×3[0.7].

## Discussion and conclusion

AML with inv(16) and deletion of 3’*CBFB* has been reported in at least 14 cases and most of the deletions were detected by FISH using *CBFB* break-apart probes [10–19]. To our knowledge, only three cases were analyzed using microarray, and of these cases, only one reported unambiguous genomic coordinates of the deletion [10, 11]. Noteworthily, all the cases analyzed with microarray, including the ones in this report, showed an additional deletion of 5’*MYH11,* suggesting that the 3’*CBFB* and 5’*MYH11* were most likely deleted together as a fusion product of inv(16) instead of being deleted separately. More than 10 *CBFB-MYH11* fusion transcripts with different sizes due to various genomic breakpoints have been reported [21, 22]. In this study, *CBFB* exons 1–5 fused with *MYH11* exons 8–42 in case 1 and *CBFB* exons 1–4 fused with *MYH11* exons 33–42 in case 2. Both are typical gene fusions in AML-associated inv(16), and the 5’*CBFB*/3’*MYH11* fusion genes are believed to be responsible for the disease of the patients [23]. However, there is no evidence of additional phenotypical effects of deletion of the 5’*MYH11*/3’*CBFB* fusion genes. Both patients in this study responded to chemotherapy well and achieved CR for multiple years. Three cancer-related genes listed in the Cancer Gene Census (CGC) in the Catalogue of Somatic Mutations in Cancer (COSMIC) were involved in the deleted regions, including *MYH11* in 16p, *CBFB* and *CTCF* in 16q (Fig. 1b) [24]. *CBFB/MYH11* gene fusion caused by inv(16) or t (16;16) results in two fusion genes, 5’*CBFB*/3’*MYH11* and 5’*MYH11*/3’*CBFB*. The former is a known pathogenic cause of AML, but the pathogenic effect of the latter is uncertain. We further reviewed reported AML cases with inv(16) and deletion of 3’*CBFB* (Table 1) and did not find significant phenotypic differences caused by the deletion of 3’*CBFB*. Of the 14 cases with clinical data available, 11 (79%) were known to achieve CR with known survival time up to 48 months at the time of report. This small cohort data appears to be in line with the CR rate (86–88%) and the five-year OS rate (50%) in AML with inv(16) [8, 25]. Taken together, the findings suggest that the 5’*MYH11*/3’*CBFB* fusion gene may play minimal roles in AML pathogenesis. *CTCF* is thought to be a tumor suppresser gene [26]. Kemp et al. reported *CTCF* haploinsufficiency destabilized DNA methylation and predisposed mice to cancer [27]. However, deletion of this gene in the current cases apparently did not result in additional phenotypic changes. Such changes, if there are any, may be less significant or overlapped with that of the AML with inv(16). Nevertheless, the significance of deletion of *CTCF* in AML with inv(16) needs to be further investigated. There are five cases with inv(16) and deletion of 3’*CBFB*, which either did not achieve CR or relapsed (Table 1). Since the deletion sizes and other genomic characteristics of these cases were undefined, it is unclear whether additional genes or other genomic elements were affected. Array analysis of similar cases may provide more insights on genomic changes and corresponding pathogenic effects in these cases.

**Table 1:** The previously reported AML cases with inv(16) and *3’CBFB* deletion

Cases	Sex	Age (yrs)	Karyotype	FISH	Microarray	CR	Survival (months)
Case 1	M	24	46,XY,inv(16)(p13.1q22)[16]/47,idem,+22[4]	nuc ish(5’CBFBx2,3’CBFB×1)(5’CBFB con 3’CBFB×1)[191/200]	arr[GRCh37] 16p13.11(15875744_16291983)× 1[0.9],16q22.1(67128019_68214140)×1[0.9],(22)×3[0.8]	Yes	36+
Case 2	M	47	48,XY,+9,inv(16)(p13.1q22),+22[20]	nuc ish(5’CBFBx2,3’CBFBx1)(5’CBFB con 3’CBFB×1)[167/200]	arr[GRCh37] (9)×3[0.7],16p13.11p12.3(15817490_16869754)×1[0.7],16q22.1(67113418_68099821)×1[0.7],(22)×3[0.7]	Yes	48+
Dawson et al.	M	30	46,XY,del(7)(q32),del(16)(q22)[10]	ish der(16)inv(16)(p13.1q22)del(16) (q22q24)(p13.3)(pter+)(p13.1)(5’CBFB+) (q22)(3’CBFB-)(q24)(qter+)[20]	arr[GRCh36] 16p13.11(15714571_16201064)× 1,16q22.1(65726391_66930693)×1	Yes	16+
Haferlach et al. case36	M	39	inv(16)(p13q22)	NA	arr16q22.1(65663366_66436096)x1	NA	NA
Haferlach et al. case37	M	70	45,X,-Y,inv(16)(p13q22)[17]/46,XY[3]	NA	arr16p13.11(15735443_16623582)x1,16q22.1(65654291_66288890)×1	NA	NA
Tirado et al.	M	13	46,XY,inv(16)(p13.1q22)[2]/46,idem,del(7)(q22q32)[16]/46,idem,t(9;22;19)(q34;q11.2;p13.1)[2]	ish inv(16)(p13.1)(5’CBFB+)(q22) (3’CBFB-)[20].nuc ish(5’CBFBx2,3’CBFB×1) (5’CBFB con 3’CBFB×1)[191/200]	ND	Yes	10+
Spencer et al.	M	8	46,XY,inv(16)(p13.1q22)[6]/46,XY,der(16)inv(16)del(16)(q22)[5]/46,XY[9]	Deletion of 3’CBFB	ND	Yes	2+
Hung et al.	M	32	46,XY,inv(16)(p13.1q22)[5]/46,idem,del(7)(q32)[9]/47,idem,+ 22[3]/48,idem,del(7)(q32),+20,+22[3]	Deletion of 3’CBFB	ND	Yes	Died
Kelly et al. case 1	F	76	46,XX,inv(16)(p13q22)[10]	Deletion of 3’CBFB	ND	Yes	4+
Kelly et al. case 2	F	20	46,XX,inv(16)(p13q22)[8]/46,XX[2]	Deletion of 3’CBFB	ND	No	NA
Kelly et al. case 3	M	68	47,XY,+8,inv(16)(p13q22)[12]/46,XY[3]	Deletion of 3’CBFB	ND	No	Died
Egan et al.	M	17	46,XY,inv(16)(p13.1q22)[17]/47,idem,+22[3]	Deletion of 3’CBFB	ND	Yes	Relapse
Kolomietz et al. case 1	NA	NA	inv(16)(p13q22)	Deletion of 3’CBFB	ND	Yes	Relapse
Kolomietz et al. case 2	NA	NA	inv(16)(p13q22)	Deletion of 3’CBFB	ND	No	Died
Pirc-Danoewinata et al.	F	18	47,XX,+22[20]^a^	Deletion of 3’CBFB	ND	Yes	42+
Batanian et al.	M	2.5	46,XY,der(16)inv(16)(p13q22)del(16)(q22)[17]/45,idem,-Y[3]	Deletion of 3’CBFB	ND	Yes	NA

*M* male, *F* female, *NA* not available, *ND* not done, *CR* complete remission

^a^5’*CBFB*/3*’MYH11* gene fusion was detected by RT-PCR

Deletion of the *3’CBFB* gene is a recurrent finding in AML with inv(16). It most likely represents deletions of the 5’*MYH11/*3’*CBFB* gene fusion from the inv(16) as a secondary change following the inversion. The 5*’CBFB*/3’*MYH11* fusion resulting from the inv(16) is a known pathogenic cause of AML, but the 5’*MYH11/*3’*CBFB* fusion may play a minimal role in AML pathogenesis. The *CTCF* gene adjacent to the 3’*CBFB* was deleted in the current cases, suggesting that deletions of *CTCF* in AML with inv(16) may not have prognostic significance either. However, potential pathogenic effects of other genes involved in extended deletion regions may not be excluded.

## Data Availability

The data used or analyzed during the current study are available from the corresponding author on reasonable request.

## Abbreviations

AML: Acute myeloid leukemia
ASCT: Autologous stem cell transplant
BM: Bone marrow
CGC: Cancer Gene Census
COSMIC: Catalogue of Somatic Mutations in Cancer
CR: Complete remission
FISH: Fluorescence in situ hybridization
Hb: Hemoglobin
ISCN: International System for Human Cytogenomic Nomenclature
MPO: Myeloperoxidase
OS: Overall survival
SNP: Single nucleotide polymorphism
WBC: White blood cell
